# Comparing Approaches to Mobile Depression Assessment for Measurement-Based Care: Prospective Study

**DOI:** 10.2196/10001

**Published:** 2018-06-19

**Authors:** Amy M Bauer, Scott A Baldwin, Joaquin A Anguera, Patricia A Areán, David C Atkins

**Affiliations:** ^1^ Department of Psychiatry and Behavioral Sciences University of Washington Seattle, WA United States; ^2^ Department of Psychology Brigham Young University Provo, UT United States; ^3^ Department of Neurology University of California, San Francisco San Francisco, CA United States

**Keywords:** Patient Health Questionnaire, depression, mobile health, symptom assessment

## Abstract

**Background:**

To inform measurement-based care, practice guidelines suggest routine symptom monitoring, often on a weekly or monthly basis. Increasingly, patient-provider contacts occur remotely (eg, by telephone and Web-based portals), and mobile health tools can now monitor depressed mood daily or more frequently. However, the reliability and utility of daily ratings are unclear.

**Objective:**

This study aimed to examine the association between a daily depressive symptom measure and the Patient Health Questionnaire-9 (PHQ-9), the most widely adopted depression self-report measure, and compare how well these 2 assessment methods predict patient outcomes.

**Methods:**

A total of 547 individuals completed smartphone-based measures, including the Patient Health Questionnaire-2 (PHQ-2) modified for daily administration, the PHQ-9, and the Sheehan Disability Scale. Multilevel factor analyses evaluated the reliability of latent depression based on the PHQ-2 (for repeated measures) between weeks 2 and 4 and its correlation with the PHQ-9 at week 4. Regression models predicted week 8 depressive symptoms and disability ratings with daily PHQ-2 and PHQ-9.

**Results:**

The daily PHQ-2 and PHQ-9 are highly reliable (range: 0.80-0.88) and highly correlated (*r*=.80). Findings were robust across demographic groups (age, gender, and ethnic minority status). Daily PHQ-2 and PHQ-9 were comparable in predicting week 8 disability and were independent predictors of week 8 depressive symptoms and disability, though the unique contribution of the PHQ-2 was small in magnitude.

**Conclusions:**

Daily completion of the PHQ-2 is a reasonable proxy for the PHQ-9 and is comparable to the PHQ-9 in predicting future outcomes. Mobile assessment methods offer researchers and clinicians reliable and valid new methods for depression assessment that may be leveraged for measurement-based depression care.

## Introduction

### Background

Practice guidelines for depression treatment call for systematic symptom monitoring to drive treatment adjustment, known as measurement-based care, an approach that improves patient outcomes [1-3]. In addition, recent value-based payment reforms have led to the creation of incentives for measurement-based care for depression by major payors such as the Centers for Medicare and Medicaid services and several large private insurers [3]. Yet, the majority of mental health providers do not use symptom rating scales, and in the few settings where scales are used routinely, they may be administered too infrequently to inform clinical decision making [3,4]. Chief among the reasons that psychiatrists and psychologists report that they do not use symptom measures is that they consider it too time-consuming or burdensome to administer measures [4,5].

For symptom measures to be clinically actionable and drive measurement-based care, the measures need to be reliable, current, interpretable, and sensitive to change [6]. In addition, experts have called for improving symptom measures by making them more brief [6]. Evidence suggests that measurement-based care is most effective when measures are completed frequently by patients in the outpatient setting, feedback is provided to both patients and clinicians, and progress is monitored over time [7]. However, these guidelines have not been fully implemented in real-world practice. Among large-scale programs identified as exemplars of measurement-based care, symptom data are often infrequently collected by providers—only when patients present to clinic or when ordered by a provider. Furthermore, feedback is usually available only to clinicians [6]. Many of these limitations can be overcome by mobile assessment tools on consumer devices that allow individuals to track their own symptoms. Wide-scale adoption of mobile mood assessment has the potential to alleviate the time burden on clinicians, support real-time symptom monitoring on a daily or more frequent basis even for people who are not presenting for clinic-based services, and may have intrinsic therapeutic value in activating patients [8,9]. However, it is unclear how brief daily ratings relate to established clinical measures and, thus, whether they may constitute useful tools for driving measurement-based care. Likewise, it is not known whether the additional information provided in daily ratings offers better prognostic information on patient outcomes that may be useful in guiding treatment decisions.

Several small-scale studies exploring associations between daily depression measures and standardized scales have yielded inconsistent results. For example, 2 small studies among patients in specialty care for depression found that brief daily measures were associated with the Patient Health Questionnaire-9 (PHQ-9) [10,11]. Likewise, a small study of patients with bipolar disorder in specialty care found that daily mood ratings were associated with a clinician-administered Hamilton Depression Rating Scale [12]. In contrast, in a community-based sample, PHQ-9 scores were not associated with 15 items indicative of depression administered by ecological momentary assessment twice daily [13]. Limitations of earlier work include the small scale of studies and the restricted range of depression symptoms in clinic-based samples.

### Objectives

In this study, the 2 primary aims were (1) to examine the association between the PHQ-9, which is the most widely adopted self-report measure that is typically given at most every 2 weeks, and a daily depressive symptom measure; and (2) to compare how these 2 assessment methods perform in predicting patient outcomes. This study, which serves as a *proof of concept* for the reliability and predictive ability of daily depression symptom measurement, is a secondary analysis of an existing dataset from a large national community-based sample that includes daily depression measures.

## Methods

### Data Source and Measures

Data came from a fully remote trial of smartphone-based depression apps in which individuals with a PHQ-9 score of 5 or greater were recruited through a Web-based interface (NCT00540865). Participants were recruited across the United States via 4 approaches: traditional approaches (written advertisements in city buses, newspapers, and Craigslist throughout the United States: 88.98%, 2601/2923); social networking methods (regular postings on sites such as Facebook and Twitter, and contextual-targeting methods to identify and directly push recruitment advertisements to potential participants, based on their Twitter and other social media comments: 0.41%; 12/2923); search engine-–based methods (Google Adwords: 0.31%, 9/2923); and unanticipated means (eg, referral or own search: 10.30%, 301/2923). Of those recruited, 1098 met eligibility criteria for the trial and were enrolled. Ethical approval was granted by the University of California San Francisco Committee for Human Research, and details of the trial, including methods for obtaining participants’ informed consent, have been previously published [14,15].

Participants completed the PHQ-9 and Sheehan Disability Scale at specified timepoints (eg, weeks 4 and 8) and received US $20 in Amazon gift vouchers for completing the assessment at each of these timepoints. The PHQ-9 is a valid measure of depression symptoms that is widely used for depression screening and treatment monitoring [16,17]. Individuals report how often over the last 2 weeks they have experienced each of 9 core symptoms of major depression. The Sheehan Disability Scale is a 3-item measure of functioning in work, social, and health domains that has been validated in medical and psychiatric populations [18,19]. Participants also self-reported depressed mood on a daily basis via a modified Patient Health Questionnaire-2 (PHQ-2). The PHQ-2, which consists of the first 2 items of the PHQ-9, is a valid screening tool for detecting depressive disorders and is sensitive to change over time [20,21]. The PHQ-2 was modified for daily administration by changing the timeframe to “yesterday” and the response options to a 5-point Likert-type scale anchored at “not at all” (1) to “most of the day” (5). Participants did not receive an incentive for completion of daily measures.

**Figure 1:**
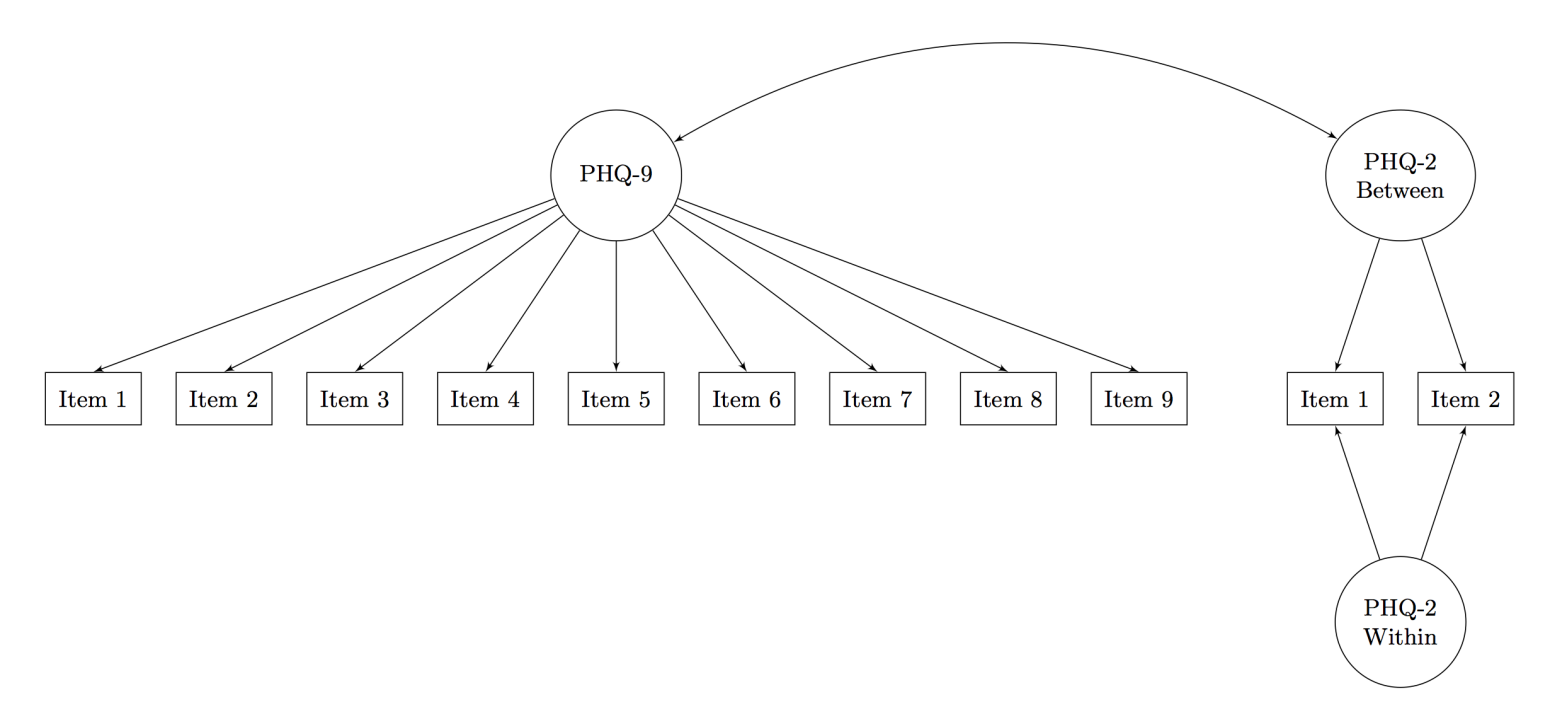
Path diagram for the factor analysis of the Patient Health Questionnaire-2 (PHQ-2) and Patient Health Questionnaire-9 (PHQ-9). The PHQ-9 was measured at week 4. The PHQ-2 was measured daily for 14 days before the PHQ-9. Rectangles represent observed variables and ovals represent latent variables. Single-headed arrows are factor loadings and double-headed arrows are correlations. Because participants responded to the PHQ-2 repeatedly over a 2-week period, within-patient variability (ie, variability day-to-day) can be separated from between-patient variability (ie, variability in average PHQ-2 scores). Residual variances were estimated in the model but not included in the path diagram.

Participants were excluded from the sample if they were missing a PHQ-9 at week 4 or if they did not have any daily PHQ-2 scores within 14 days preceding their week 4 PHQ-9 score. In addition, participants were excluded from the regression analysis if they were missing the PHQ-9 or Sheehan score at week 8 (see below for a description of the analyses).

### Data Analysis

To address our first aim, we compared week 4 PHQ-9 with all PHQ-2 scores within 14 days preceding the week 4 PHQ-9 (the timeframe for PHQ-9 response). Specifically, we fit a multilevel confirmatory factor analysis [22] for the PHQ-2 repeated measures and single-level confirmatory factor analysis for the PHQ-9 to evaluate the reliability and correlation of latent depression across these measures (see Figure 1 for a path diagram). The PHQ-2 and PHQ-9 models were estimated in a single analysis, producing a single model and set of fit statistics. We computed reliability of the PHQ-9, between-persons reliability of the PHQ-2 (ie, average depression over a given time period for a specific person), within-persons reliability of the PHQ-2 (ie, reliability of day-to-day variability in depression for a specific person), and the correlation between the 2 latent depression scores (similar to comparing the PHQ-9 with the average of the PHQ-2 over 2 weeks).

We also used a multiple group confirmatory factor analysis to examine differences in reliability and the correlation between the PHQ-2 and PHQ-9 for groups based on age, gender, and racial or ethnic minority status. Separate multiple group models were used to compare the following groups: racial or ethnic minority versus white, men versus women, and age 55+ years versus <55 years. The multiple group models compared an unconstrained model with a constrained model. In the unconstrained model, all factor loadings, intercepts, and residuals were uniquely estimated in each group. In the constrained model, all factor loadings, intercepts, and residuals were constrained to be equal across groups. Given that reliability is a function of factor loadings and residual variances [23] and that it is generally inappropriate to constrain residuals but not intercepts across groups [23], we chose to constrain all parameters.

For our second aim, we evaluated how well each depression measure (PHQ-9 at week 4 and daily PHQ-2 from weeks 2 to 4) predicted PHQ-9 and the Sheehan Disability scale at week 8. One potential advantage of repeatedly administering the PHQ-2 is that the repeated measures can be summarized with a variety of indicators. For example, in addition to the mean PHQ-2, we can examine the predictive ability of parameters such as the score trajectory over time (slope) or highest PHQ-2 score (maximum). Therefore, to construct predictors, we summarized the daily PHQ-2 using the mean daily value, SD, minimum, maximum, and linear slope. The first primary model included the PHQ-9 and PHQ-2 mean as predictors and the second included the PHQ-9; PHQ-2 mean; and the PHQ-2 SD, minimum, maximum, and slope as predictors. We used seemingly unrelated regression [24] to estimate the models, which is a multivariate regression approach that allowed to simultaneously fit the regression model for both week 8 PHQ-9 and the Sheehan Disability scale.

## Results

### Participants

Among 1098 adult participants, 545 individuals completed the PHQ-9 at week 4 and at least one PHQ-2 in the preceding 2 weeks. Of these, 3 individuals were missing demographic data and therefore could not be included in factor analyses with demographic data. The final dataset has 2992 observations on 545 participants. Participants completed an average of 5.5 daily measurements (see Table 1 for distribution of responses). Among the final sample, 78.3% (427/545) were women and 38.1% (208/545) were identified as racial or ethnic minorities, which is similar to national rate of 39% [25]. The average age was 33 years (SD 11) and 6.1% (33/545) participants were 55 years of age or older. The average PHQ-9 score at baseline was 13.9 (SD 5.0) and 77.8% (424/545) had a PHQ-9 score of 10 or greater. Participants were predominantly employed (68.3%, 372/545) and most (60.0%, 327/545) had at least a bachelor’s degree. Income was widely distributed (≤$20,000: 22.6% [123/545]; $20,000-$50,000: 18.0%, [98/545]; $50,000-$80,000: 10.0% [55/545]; >$80,000: 4.4% [24/545]; missing: 44.4% [242/545]). A minority (27.3%, 149/545) were married or partnered.

### Reliability

Table 2 presents the results of the factor analysis of the week 4 data (see Figure 1 for a path diagram). To identify the model, we constrained the mean of each latent variable to 0 and the variance to 1. Given that the PHQ-2 had only 2 indicators, we constrained the loadings at the between level to be equal and the loadings at the within level to be equal. Overall fit of the model was good as indicated by a comparative fit index=0.94, Tucker-Lewis index=0.93, and root mean square error of approximation=0.04. Factor loadings were generally strong for the PHQ-9, with the weakest loadings being for items 8 and 9 (psychomotor symptoms and suicidal ideation). Loadings for the PHQ-2 were strong at both levels.

**Figure 2:**
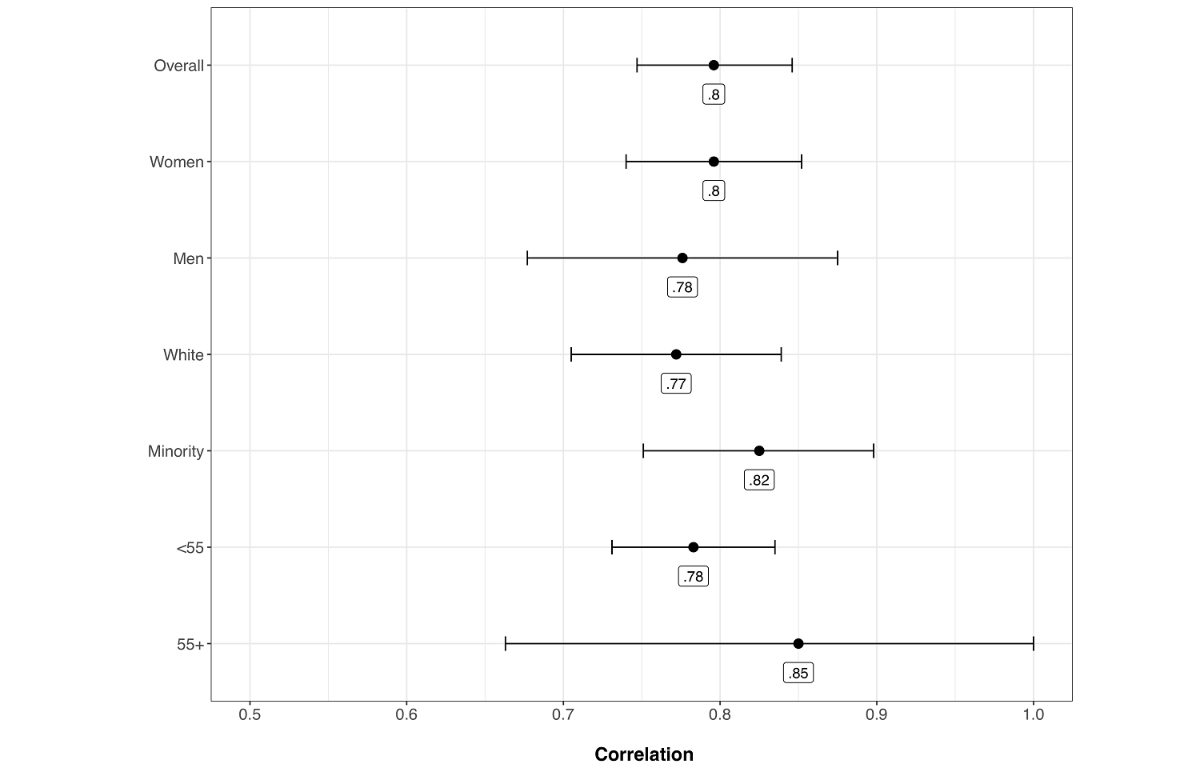
Correlation between Patient Health Questionnaire-2 and Patient Health Questionnaire-9. Interval estimates are 95% CIs. Point estimates are rounded to 2 digits. PHQ: Patient Health Questionnaire; <55 refers to participants under the age of 55 years; 55+ refers to participants aged 55 years or older.

**Figure 3:**
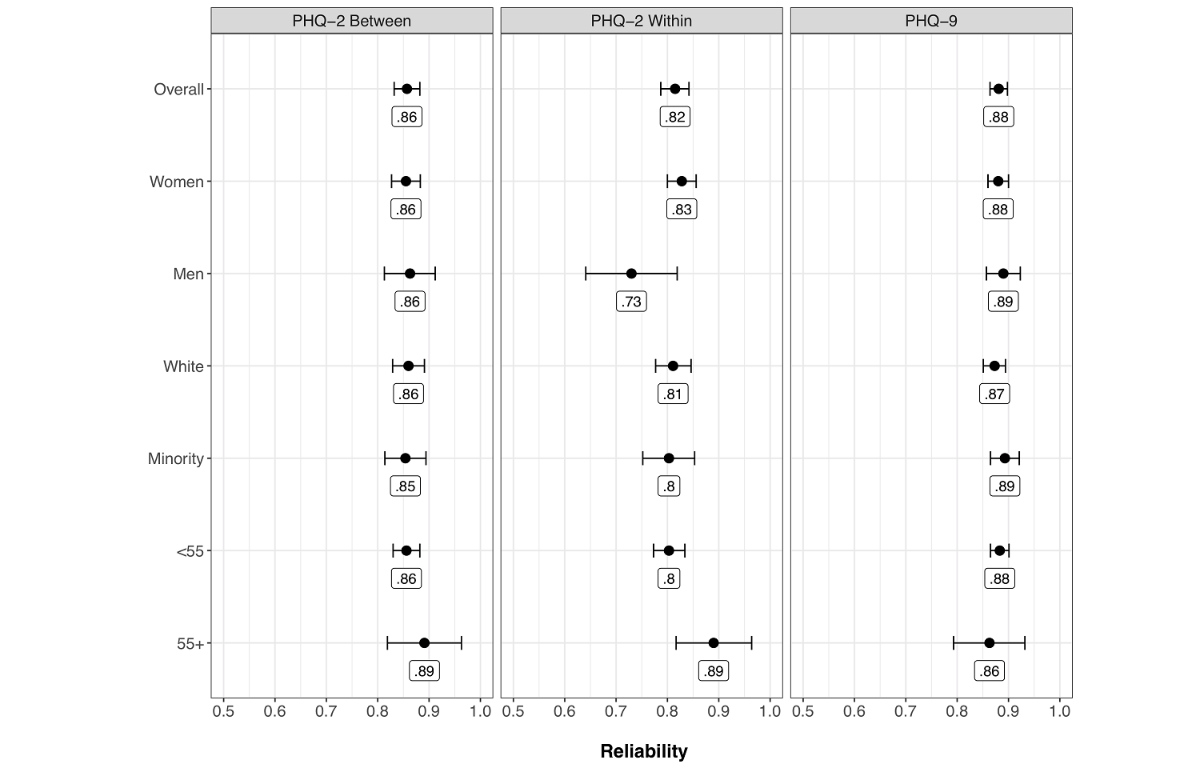
Reliability estimates. Interval estimates are 95% CIs. Point estimates are rounded to 2 digits. PHQ: Patient Health Questionnaire; <55 refers to participants under the age of 55 years; 55+ refers to participants aged 55 years or older.

The PHQ-9 and between-persons PHQ-2 were strongly correlated, *r*=.8 (*P*<.001; see Figure 2). Reliability was also high. Specifically, reliability for the PHQ-2 was estimated as 0.82 (within-persons) and 0.86 (between-persons). Consequently, day-to-day variability in PHQ-2 and the PHQ-2 average over 2 weeks were reliable. Reliability for the PHQ-9 was estimated as 0.88 (see Figure 3 and Table 2).

For all but the minority versus white group comparisons, the unconstrained model fits better than the constrained model: men versus women, χ^2^_32_=84.0, *P*<.001; 55+ years versus <55 years, χ^2^_32_=57.8, *P*=.004; minority versus white, χ^2^_32_=38.0, *P*=.21. This suggests that men and women and participants who are aged 55 years and above and participants who are <55 years have a *statistically* different factor loadings, intercepts, residuals, and reliabilities. In contrast, participants from racial or ethnic minority groups and white participants did not statistically differ.

Figure 2 shows the estimated correlation by group between the PHQ-9 and PHQ-2 based on the unconstrained model. Figure 3 shows the estimated reliabilities by group based on the unconstrained model. The differences in the correlations and reliabilities were generally small and likely of little practical significance. The most notable difference is between men and women for the within-person PHQ-2 reliability. Specifically, men had a lower estimated reliability but also more uncertainty (ie, wider CI) than women, which is likely because of the fact that there were fewer men than women in the sample.

### Predicting Future Functioning

Week 4 PHQ-2 and PHQ-9 were statistically significant predictors of both the PHQ-9 and the Sheehan Disability Scale (SDS) at week 8 (Table 3). This was true when the week 4 predictors were entered into the regression alone and together (compare Models 1 and 2 with Model 3 in Table 3). *R*^2^ for Model 1, where PHQ-9 at week 4 was the only predictor, was 0.49 (PHQ-9 week 8) and 0.37 (SDS week 8). *R*^2^ for Model 3, where both week 4 PHQ-9 and PHQ-2 were predictors, was 0.50 (PHQ-9 week 8) and 0.41 (SDS week 8). This suggests that PHQ-2 does not provide much predictive information above and beyond the PHQ-9 when predicting future PHQ-9 values. When predicting future functional disability with the SDS, a more global measure of outcomes, the PHQ-2 does add somewhat to the predictive information of the PHQ-9.

**Table 1:** Distribution of daily Patient Health Questionnaire-2 (PHQ-2) ratings completed.

Number of daily ratings submitted	Participants, n (%)	Cumulative, n (% )
1	40 (7.3)	40 (7.3)
2	22 (4.0)	62 (11.4)
3	27 (5.0)	89 (16.3)
4	45 (8.3)	134 (24.6)
5	79 (14.5)	213 (39.1)
6	109 (20.0)	322 (59.1)
7	199 (36.5)	521 (95.6)
8	18 (3.3)	539 (98.9)
9	2 (0.4)	541 (99.3)
10	2 (0.4)	543 (99.6)
11	1 (0.2)	544 (99.8)
12	1 (0.2)	545 (100.0)
13	0 (0.0)	545 (100.0)
14	0 (0.0)	545 (100.0)

**Table 2:** Factor analysis at week 4.

Measure		Loadings^a^	Intercept	Variance^b^	Reliability
**PHQ-9^c^**			0.0^d^	1.0^d^	0.88
	Item 1	0.60	1.07	0.20	
	Item 2	0.63	1.12	0.22	
	Item 3	0.59	1.25	0.53	
	Item 4	0.65	1.39	0.40	
	Item 5	0.65	1.07	0.58	
	Item 6	0.70	1.03	0.44	
	Item 7	0.56	0.90	0.45	
	Item 8	0.34	0.34	0.32	
	Item 9	0.37	0.34	0.36	
**PHQ-2^e^ between**			0.0^d^	1.0^d^	0.86
	Item 1	0.85^d^	2.20	0.05	
	Item 2	0.85^d^	2.20	0.05	
**PHQ-2 within**			0.0^d^	1.0^d^	0.82
	Item 1	0.66^d^	—	0.20	
	Item 2	0.66^d^	—	0.20	
Participants (N)		545			
Comparative fit index		0.94			
Tucker-Lewis index		0.93			
Root mean square error of approximation		0.04			

^a^All estimated coefficients were statistically significant.

^b^Variances on rows with items are residual variances and variances on other rows are variances.

^c^PHQ-9: Patient Health Questionnaire-9.

^d^Constrained for identification of latent variables.

^e^PHQ-2: Patient Health Questionnaire-2.

**Table 3:** Prediction of week 8 outcomes based on depression scores at week 4.

Outcome^a^ and predictor^b,c^		Model 1		Model 2			Model 3			Model 4		
		Coefficient^d^	*P* value		Coefficient^d^	*P* value		Coefficient^d^	*P* value		Coefficient^d^	*P* value
**PHQ-9^e^**												
	PHQ-9 mean	0.7	<.001		—			0.6	<.001		—	
	PHQ-2^f^ mean	—			0.4	<.001		0.1	.001		0.3	.002
	PHQ-2 slope	—			—			—			0.5	.43
	PHQ-2 max^g^	—			—			—			−0.1	.22
	PHQ-2 min^h^	—			—			—			0.2	.11
	PHQ-2 SD	—			—			—			0.5	.04
	*R* ^2^	0.49			0.34			0.50			0.37	
**Sheehan Disability Scale**												
	PHQ-9 mean	2.7	<.001		—			1.9	<.001		—	
	PHQ-2 mean	—			1.7	<.001		0.8	<.001		1.5	.001
	PHQ-2 slope	—			—			—			0.8	.77
	PHQ-2 max	—			—			—			0.6	.16
	PHQ-2 min	—			—			—			−0.4	.43
	PHQ-2 SD	—			—			—			−1.2	.26
	*R* ^2^	0.37			0.32			0.41			0.34	
	N^i^	352			362			348			351	

^a^Outcomes were all measured at week 8.

^b^Predictors were all measured at week 4.

^c^Each model has a different set of predictors. The coefficient and *P* values are listed only for those predictors that were included in the Model.

^d^Unstandardized regression coefficients are reported.

^e^PHQ-9: Patient Health Questionnaire-9.

^f^PHQ-2: Patient Health Questionnaire-2.

^g^Max: maximum.

^h^Min: minimum.

^i^N: Number of participants.

One potential advantage of daily symptom monitoring is that this allows patients’ scores to be characterized in more nuanced ways in addition to the daily average, such as the slope over 2 weeks or the minimum or maximum value. Model 4 shows that slope, maximum, minimum, and SD of the PHQ-2 over the 2 weeks preceding week 4 were not significant predictors of either the PHQ-9 or the SDS at week 8, suggesting no additional predictive ability of these features beyond the daily average PHQ-2.

## Discussion

### Principal Findings

Our findings establish that both the daily PHQ-2 mean and daily PHQ-2 variability are reliable measures of depressive symptoms. The daily PHQ-2 mean is closely correlated with the PHQ-9, the most commonly used measure for assessing depressive symptom severity. These findings hold across a range of demographic groups. As such, we have identified that a brief daily measure can provide current, accurate information on depressive symptom status, thus fulfilling several of the qualities needed for a measure to be clinically actionable and inform measurement-based care [6].

Our findings further demonstrate that daily PHQ-2 mean between weeks 2 and 4 is a strong predictor of depressive symptoms and of overall functioning at week 8 that is independent of the PHQ-9. Although it is an independent predictor of week 8 outcomes, the magnitude of the independent contribution of the PHQ-2 is small when a PHQ-9 is obtained at week 4. Likewise, despite demonstrating that daily PHQ-2 variability is a reliable measure, none of the daily indicators we examined (minimum, maximum, slope, and SD) improved prediction of week 8 outcomes above and beyond the daily PHQ-2 mean. Therefore, it appears that the predictive value of the PHQ-2 in this sample is related to the stable information obtained from the average of the PHQ-2 across the 2 weeks and not the repeated assessments. The daily PHQ-2 is somewhat less strongly predictive of week 8 depressive symptoms than the week 4 PHQ-9, although this is not surprising given that week 8 depressive symptoms were measured with the PHQ-9. Importantly, when we examined a more global outcome measure at week 8, the Sheehan Disability Scale, the daily PHQ-2 performs comparably to the week 4 PHQ-9 in predicting the week 8 outcomes. Therefore, the daily PHQ-2 may serve as a proxy measure that can reasonably substitute for the PHQ-9. Given the favorable psychometric properties and strong predictive ability of each measure, researchers and clinicians may wish to consider response rate when selecting a mobile depression measure. In this sample, the response rate for the daily PHQ-2 was greater than the response rate for the PHQ-9 at all timepoints [26], further supporting the utility of the daily PHQ-2. However, it is possible that the response rates in this sample may differ from those that would be obtained in a clinical sample.

### Limitations

Our findings are based on a large national sample of community-dwelling individuals who reported a range of depressive symptom scores, which represent a strength for the psychometric analyses we conducted. However, certain limitations also apply. The sample consisted of moderately depressed individuals, 78.3% (427/545) of whom score 10 or above on the PHQ-9. This restricted the response range of participants; however, it is also similar to the scores that would be observed among patients in clinical settings, and the reliability of the PHQ-9 in this study (0.88) is similar to reported values ranging from 0.79 to 0.86 [27]. The factor analysis models were based only on PHQ-9 scores at a single timepoint (week 4) and not all participants completed all daily PHQ-2 measures. Data came from a broad community-based sample that increased generalizability; however, detailed clinical or diagnostic information was not available to characterize participants. Although it is likely that individuals who volunteered to participate in this study differed from nonparticipants in certain characteristics, it is unknown to what extent these differences would have affected the psychometric properties of the measures or the relationship between the daily PHQ-2 measure and the PHQ-9 or disability scores. Similarly, we were unable to separate variability due to completing an assessment from the variability due to responding to these specific questions about depression. Future research that seeks to separate these sources of variability could have participants respond to depression items as well as neutral items to determine which responses are predictive of future symptoms.

We analyzed the modified PHQ-2 as the daily measure based on the availability of this measure in the existing dataset. Although our findings support the use of a daily PHQ-2 for monitoring depressive symptoms, these findings may not entirely translate to other daily mood measures. Our findings can serve as a *proof of concept* for brief daily depression symptom measurement; however, future studies should assess other measures. Despite receiving daily notifications, participants completed an average of 5.5 daily ratings. This study did not assess patients’ experiences with daily depression monitoring; however, previous research does support the acceptability of monitoring depressive symptoms on a daily basis [28]. In our ongoing work with a weekly symptom monitoring app, one of the most common requests from patients has been for the addition of a daily mood measure [29]. Although, on average, participants completed fewer than half of the daily ratings, this represents completion of a symptom measure every 2 to 3 days, which is considerably more frequent than clinic-based assessment. Treatment dropout is a serious concern with clinic-based services, given that the modal number of visits for depressed patients receiving behavioral interventions is 1 [30,31]. Daily mobile symptom monitoring may serve to promote better engagement in care, and future research in clinical samples should examine its potential to promote retention among patients who may otherwise discontinue services prematurely.

### Future Directions

Our findings support the use of a daily PHQ-2 as an alternative to the PHQ-9 for monitoring depressive symptoms. Our finding that daily average PHQ-2 is an independent predictor of week 8 depressive symptoms and functioning demonstrates that a daily measure does provide additional information. Although we found that daily depressive symptom variability as measured by the PHQ-2 did not substantially improve prediction of week 8 outcomes, it is plausible that indicators of daily variability in depressive symptoms may have greater utility at certain phases of treatment, for example, as indicators of incomplete treatment response that may be associated with higher risk of relapse. Future research should examine other types of brief mobile mood measures and passively collected behavioral indicators to evaluate how such measures can be combined with or substitute for self-report measures and whether novel assessment (eg, Photographic Affect Meter [32]) may be more engaging to patients and facilitate long-term self-monitoring. This study serves as a model for researchers examining other brief mobile assessment methods, and future work should link daily mood assessments with clinical information to determine the clinical utility of new assessment methods and to determine the optimal frequency of measurement.

### Conclusions

This study represents an important step in establishing a daily depressive symptom measure to drive measurement-based care. Mobile assessment methods that are convenient to administer, reliable, and predict functioning may facilitate the adoption of measurement-based depression care and improve the quality of care and health outcomes. Because optimal outcomes from measurement-based care are achieved when results of such measures are provided to clinicians and incorporated in a structured manner into clinical encounters [7,33], future research should explore the incorporation of daily mood measures within the context of comprehensive measurement feedback systems.

## Abbreviations

PHQ-2: Patient Health Questionnaire-2
PHQ-9: Patient Health Questionnaire-9
SDS: Sheehan Disability Scale
UCSF: University of California, San Francisco
